# Editorial: Emerging and reemerging neglected tropical diseases: their epidemiology, transmission, mitigation, and vaccines and chemotherapeutics advancements, volume II

**DOI:** 10.3389/fphar.2026.1864825

**Published:** 2026-06-05

**Authors:** Ranjan K. Mohapatra, Snehasish Mishra, Venkataramana Kandi, Nrusingh P. Mohapatra, Chandra Sekhar Sirka

**Affiliations:** 1 Department of Chemistry, Government College of Engineering, Keonjhar, Odisha, India; 2 School of Biotechnology, KIIT Deemed-to-be-University, Bhubaneswar, Odisha, India; 3 Department of Microbiology, Prathima Institute of Medical Sciences, Karimnagar, India; 4 Nikoni Pharmaceuticals LLC, Maryland Heights, MO, United States; 5 Liponyx Pharmaceuticals Ltd., Bhubaneswar, Odisha, India; 6 Department of Dermatology, Venereology, All India Institute of Medical Sciences, Bhubaneswar, Odisha, India

**Keywords:** epidemiology, mitigation, NTDs, transmission, vaccines and chemotherapeutics advancements

## Introduction

Neglected tropical diseases (NTDs) remain a major global health challenge that affects more than 1.5 billion people across nations. Diseases like lymphatic filariasis, leishmaniasis, schistosomiasis, and trachoma, to mention a few, impact impoverished communities that lack clean water, sanitation and healthcare disproportionately. The World Health Organisation (WHO) recognised 21 NTDs, which collectively cause over 200,000 deaths annually and contribute to millions of disability-adjusted life years (DALYs), outdoing the Human Immunodeficiency virus (HIV)/Acquired Immunodeficiency Syndrome (AIDS), tuberculosis and malaria burden. Termed as ‘neglected*’*, many of these diseases have been wiped out from most of the developed societies, while still persisting in the poor and most marginalised ones (Álvarez-Hernández et al., 2020). Despite the progress, including eliminating the guinea worm disease in most regions and reducing trachoma and yaws significantly, many NTDs still persist in Africa, Southeast Asia and Latin America (Srivastava et al., 2023; Ca et al., 2024; Mohapatra et al., 2024). Mass drug administration programmes have reached billions worldwide, yet institutional funding remains critically low, with only 0.6% of the global health development assistance directed toward NTDs. WHO’s 2030 roadmap sets ambitious targets of reducing the number of people requiring NTD interventions by 90%, cutting DALYs by 75%, and achieving eradication of dracunculiasis and yaws (WHO, 2025). However, challenges of underreporting, weak surveillance, climate change and disruptions due to Coronavirus Disease 2019 (COVID-19) pandemic continue to hinder the progress.

This Editorial synthesises recent research and reviews focusing on NTDs, zoonotic infections and viral pathogens. It underscores the necessity of integrated strategies in global health and highlights the underappreciated significance of mosquitoes in swine farm biosecurity. Structural housing measures in animal farming could be pivotal, as they would facilitate or prevent vector’s entry, impacting both disease transmission and animal productivity. This Research Topic reviews the global NTD burden that affects over a billion humans, primarily in the impoverished regions, and also emphasises the urgent need to innovate affordable and rapid diagnostic tools and techniques. While technological advances such as clustered regularly interspaced short palindromic repeats (CRISPR)-based diagnostics, biosensors and artificial intelligence hold great promises, their use remains limited.

This Editorial also discusses therapeutic strategies, including mass drug administration, and outlines the challenges posed by drug resistance and supply chain management issues. The 2021–2030 roadmap of WHO sets forth ambitious elimination targets, emphasising the importance of One Health frameworks, public–private partnerships, and sustainable financing (WHO, 2025). It also calls for multidisciplinary solutions encompassing improved diagnostics, novel therapeutics, vector control and international collaborations.

Hanthorn et al. stress the necessity to integrate approaches that combine microbial and vector-focused biosecurity, particularly within swine housing. Current pig farming biosecurity protocols largely focus on microbial pathogens, often neglecting the significant role of mosquitoes and other insect vectors. Their study demonstrates that the design and structural components of swine farms, like walls, ventilation systems and openings serve either as barriers or as entry points for mosquitoes, with direct implications for disease transmission, animal wellbeing and the overall productivity. Mosquito infestations not only facilitate the spread of vector-borne diseases affecting both pigs and humans, but also lead to mechanical damage, irritation, secondary infections, stress and reduced productivity. Effective mosquito control could additionally minimise the burden of other insect pests, contributing to improved animal wellbeing and reduced production losses. Structural design aimed at preventing mosquito entry is integral to maintaining robust farm biosecurity and overall animal health.

Kumar et al. highlighted that NTDs impacted over a billion globally, predominantly in the poverty-laden tropical and subtropical regions. These diseases inflict chronic disability, social stigma and economic hardship, thereby perpetuating the poverty cycle. Transmission dynamics are complex, involving interactions among humans, animals, and mosquito, fly and snail as vectors. The aggravating factors included climate change, population displacement, inadequate sanitation and fragile healthcare systems. Among the classified 21 NTDs, as recognised by the WHO, are leishmaniasis, Chagas disease, lymphatic filariasis, schistosomiasis and snakebite envenoming. Conventional diagnostics—like microscopy and immunoassays—are often slow, invasive and unreliable, notably lacking rapidity, affordability and point-of-care testing option that are suitable for low-resource settings. Though advanced techniques like loop-mediated isothermal amplification (LAMP), CRISPR-based assays, biosensors and AI-powered diagnostics exist, their implementation remains limited. The mainstay therapy for several NTDs is mass drug administration (MDA), as witnessed in managing lymphatic filariasis, trachoma and helminth infections. Diseases like leishmaniasis, Chagas disease, and Buruli ulcer need specialised treatments. Major obstacles include drug resistance, high treatment costs and constrained supply chains. The authors emphasise that innovation in diagnostics is the most critical gap. Efforts to control and eliminate NTDs will fail without reliable and accessible diagnostic tools, regardless of the therapeutic advances. A holistic approach that combines diagnostic innovation, effective treatments, climate adaptation and community engagement is vital for sustainable progress.

Sahu et al. reviewed the current challenges for Chikungunya virus (CHIKV), another arthropod-borne viral disease that spreads to humans through mosquito bite. The disease was endemic to the Indian Ocean region and affected millions of people until 2004. It has progressively expanded to other nonendemic regions in the Middle-East, Europe and the Pacific. Conventional vector control measures lack efficacy and highlights the need of innovative control measures and devising novel diagnostic techniques. Further, as existing assays vary on sensitivity and specificity, mis-reporting and under-reporting of CHIKV cases is yet another challenge. The authors also suggest strategies to deploy novel sustainable measures in resource-limited settings to address infection and disease transmission effectively.

Kumar et al. in their opinion piece, focus on cutaneous leishmaniasis (CL), a disease caused by protozoan parasite of the genus *Leishmania*, transmitted by infected sandfly. CL represents a significant public health concern, especially in the endemic regions across Middle-East, South America, Africa and Asia. Altered environment, urbanisation, migration, and conflicts contribute to the spread of the disease, and complicate the control efforts. The diagnosis was challenging due to diverse clinical presentations ranging from self-resolving localised lesions to chronic, disfiguring forms. While conventional diagnostic techniques like microscopy, culture and serology were still used, molecular diagnostic techniques like polymerase chain reaction (PCR) are increasingly critical for species-level identification and treatment plans. Managing CL was complicated due to the effectiveness of Leishmania drugs across species, geographic location and the host/reservoir factors. Pentavalentantimonials, Amphotericin B and Miltefosine traditional treatments were less effective due to resistance and toxicity. Localised therapies (like cryotherapy, thermotherapy and topical agents) were good for mild cases, and systemic drugs were reserved for severe or refractory illness. The need for safer, more effective and affordable treatment options, and integrated strategies that incorporate vector control, surveillance and patient management was urgent. Research highlighted the value of host immune responses in disease outcome, spurring interest in immunomodulatory therapies and vaccine development. Addressing CL needs multidisciplinary efforts involving epidemiology, diagnostics, novel therapies and public health interventions to reduce the disease burden and prevent long-term disabilities.

Han et al. reviewed the public health impact of flaviviruses, including dengue, Japanese encephalitis, West Nile, yellow fever and Zika. These pathogens are significant global threats due to their ability to cause neuroinvasive diseases, haemorrhagic fevers, and congenital syndromes. The interferon (IFN) system, especially type I and type III IFNs, is central to establishing antivirals and safeguarding critical barriers such as the placenta and blood-brain barrier. Nonetheless, flaviviruses have evolved diverse strategies to evade bodily defences by targeting key IFN induction and signalling pathways components, like retinoic acid-inducible gene I (RIG-I), melanoma differentiation-associated gene 5 (MDA5), mitochondrial antiviral signalling (MAVS), TANK-binding kinase 1 (TBK1), stimulator of interferon genes (STING), interferon regulatory factor 3 (IRF3), signal transducer and activator of transcription (STAT) 1, STAT2, and the interferon (IFN)-*α*/*β* receptor (IFNAR1) pathway. Viral proteins like the non-structural protein 1 (NS1), NS4A/B, NS5, disrupt IFN responses at multiple levels and enhance viral replication and persistence. At host barrier sites, IFNs restrict viral replication but may also contribute to immunopathology, exemplified by Zika virus that could cross the placenta and blood–brain barriers while suppressing the production of IFN. Similar mechanisms are manifested by Japanese encephalitis and West Nile viruses, which exploit ‘Trojan horse’ strategies to invade the central nervous system (CNS). Some pathogens have adopted the mechanisms to evade detection by the immune system to invade the host. ‘Trojan Horse Theory’ in immunology describes the ability of some microbes, using the immune system cells as vectors, to escape the immune action. Trojan Horse is the process used by pathogens to exploit the phagocytic cells—neutrophils and macrophages—which are among the first to respond at the infection site. These cells possess microbicidal mechanisms designed to eliminate the invading microbe and help control infection, relying on the release of cytokines, chemokines, reactive oxygen species (ROS) and IFNs. However, pathogens evolve to evade or suppress these defense responses. By doing so, they survive within the host and disseminate to target tissues where they replicate while remaining shielded from the actions of the immune system. In terms of therapy, targeting IFN pathways is promising. Animal studies confirmed the necessity of intact IFN signalling to limit neuroinvasion, and preliminary clinical data suggest interferon therapy could alleviate flavivirus diseases. However, recent insights are derived from the *in vitro* studies that highlight the need for more robust *in vivo* evidence. The review underscored the evolutionary arms race between flaviviruses and host immunity, as well as the therapeutic potential to harness IFN pathways.

Lee et al. provided a comprehensive review of neurocysticercosis (NCC) by *Taeniasolium* larvae, the most prevalent parasitic infection of the CNS and is a leading cause of acquired epilepsy worldwide. NCC is now recognised as a complex neurological condition requiring multidisciplinary care. Clinical spectra of NCC have broadened significantly and include psychiatric symptoms, cognitive impairment and atypical radiological findings in addition to traditional presentations like seizures and focal neurological deficits. This diversity reflects the differences in parasite burden, lesion location, host immune response and disease stage. Advances in diagnostics have improved sensitivity and specificity in detecting NCC, with neuroimaging modalities including the magnetic resonance imaging (MRI) and computed tomography (CT) remaining central, complemented by serological, antigen-detection and molecular assays. These tools enable better differentiation between active and calcified lesions and help develop informed treatment strategies. Integrating imaging with immunological and molecular diagnostics is crucial to define a case accurately. Therapeutic approaches are shifting from standard antiparasitic regimens (albendazole and praziquantel) towards more individualised strategies, including adjunctive corticosteroids, antiepileptic drugs, immunomodulators, combination therapies and targeted interventions. Emerging evidence supports tailored treatment for lesion type, number and stage, balancing parasite clearance with inflammation control to minimise neurological complications. The authors opined that continued research to refine management, address drug resistance and improve access to care in endemic regions was needed.

## Conclusion

**FIGURE 1:**
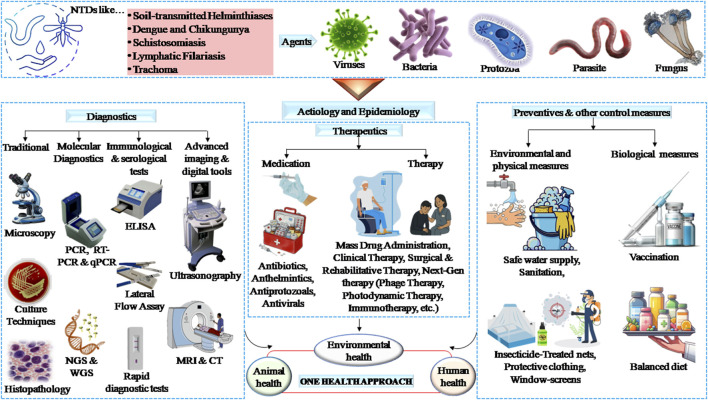
The paradigm of Neglected Tropical Diseases (MRI: magnetic resonance imaging; CT: computed tomography; PCR: polymerase chain reaction).

The contributions highlighted in this Editorial collectively emphasise the urgent need for integrated, multidisciplinary strategies to address complex infectious diseases challenges. From vector-borne threats in livestock production to NTDs, cutaneous leishmaniasis, flavivirus infections, Chikungunya infections and neurocysticercosis, the studies reviewed and illustrated both the diversity of pathogens and the commonalities in their impact on human and animal health. Several overarching themes emerge. First, diagnostic innovation remains the most pressing gap, with current tools often slow, invasive or inaccessible in resource-limited settings. Advances in molecular assays, biosensors and AI-driven platforms hold promise but require broader adoption. Second, therapeutic strategies are evolving, with traditional regimens increasingly complemented by novel nanotechnology-based delivery, immunomodulators and combination therapy approaches. These innovations must be matched by efforts to overcome drug resistance, cost barriers and supply-chain limitations. Third, integrated frameworks including ‘One Health’ approaches (Figure 1), vector control and biosecurity measures were essential to reduce transmission, improve patient outcomes and strengthen resilience against emerging threats. Finally, the expanding clinical spectrum of many infections underscores the need for heightened awareness among clinicians, improved surveillance, and tailored management strategies. Taken together, these insights reinforce that progresses against infectious diseases depend not only on scientific advancements but also on sustained global cooperation, policy reforms, and investment in health systems. By aligning diagnostics, therapeutics and public health interventions within a holistic framework, the ambitious elimination and control targets set for the coming decade could become an achievable reality.
